# Dentists in the Army and Navy

**Published:** 1899-01

**Authors:** 


### Dentists in the Army and Navy.

This subject has been under consideration by the dental profession for many years, indeed there are very few societies of any considerable membership that has not considered, more or less fully, this question. Physicians have also given some attention to it; Government officials of the army and navy have occasionally had their attention called to it. The desirability and importance of dentists in the army and navy has been realized by both officers and privates, who have, in innumerable cases, endured great suffering from diseased teeth while in the performance of military duties. The Government in the selection of volunteers is careful to give strict attention to the teeth of applicants, and if these are in any degree defective, even to the loss of a single tooth in the front of the mouth, the applicants are rejected. So far as the occurrence of diseases of the teeth and the results from these are concerned, the Government and its officials seems to take no thought. Men in the army and navy are far more liable to diseases of the teeth than those in civil life, and they are as much entitled to protection in this matter as those in any walk of life, yes, far more so; they are so because of the interruption in the performance of their work; they are so because their comfort and welfare are matters of public interest.

A law for the introduction of dentists into the army and navy has in the past years been regarded as not within the probability of attainment, probably in the near future; but from recent movements light seems to be breaking in this direction. A bill for the reorganization of the army and navy is now under consideration, and among the things to be changed, and attained is the provision for a new core of educated dentists, one hundred strong, ranking as first lieutenants, mounted. This would seem as though this subject was commanding the earnest attention of some at least who are in authority. While our professional brethren are not to be excited in regard to this movement, it is well that they should have it in mind, and study the question as thoroughly as possible, and especially when called upon, to be able to give intelligent information to any who may be searching for it and would be interested to have it.

Another point on this question that should receive attention is seen in the statement, "a new core of *educated* dentists." Of course, entering such a position, and especially in the line as here indicated, would be by examination. This examination would doubtless pertain not only to the professional qualifications of the proposed incumbent but to their general literary and educational training; in this respect the attainments should be equal to those required of the surgeon.

This revised bill, we understand, is in the hands of a

Committee, and of course may be modified or changed, but it is certainly encouraging that the subject is receiving the attention and consideration of those in authority, and we earnestly trust that it may result in establishing in the army and navy this long needed and very desirable branch of service.

